# Development of a peer support intervention to improve the experience and outcomes of discharge from inpatient mental health care: the role of experiential knowledge in a coproduced approach

**DOI:** 10.1186/s13104-021-05735-0

**Published:** 2021-08-21

**Authors:** Jacqueline Marks, Rhiannon Foster, Sarah Louise Gibson, Alan Simpson, Miles Rinaldi, Julie Repper, Jessica Worner, Shalini Patel, Mike Lucock, Michael Ussher, Sarah White, Lucy Goldsmith, Sally Barlow, Steve Gillard

**Affiliations:** 1 St George’s, University of London, London, UK; 2 Kings’ College London, London, UK; 3 South West London & St George’s Mental Health NHS Trust, London, UK; 4 Nordland Hospital Trust, Centre for Work and Mental Health, Bodø, Norway; 5 Nottinghamshire Healthcare NHS Foundation Trust, Nottingham, UK; 6 Together for Mental Wellbeing, London, UK; 7 University of Huddersfield, Huddersfield, UK; 8 City, University of London, London, UK

**Keywords:** Peer support, Mental health services, Randomised controlled trial, Complex intervention, Psychosocial interventions, Intervention development, Coproduction, Experiential knowledge

## Abstract

**Objectives:**

Peer support is rapidly being introduced into mental health services internationally, yet peer support interventions are often poorly described, limiting the usefulness of research in informing policy and practice. This paper reports the development of a peer support intervention that aims to improve outcomes of discharge from inpatient to community mental health care. People with experiential knowledge of using mental health services—peer workers and service user researchers—were involved in all stages of developing the intervention: generating intervention components; producing the intervention handbook; piloting the intervention.

**Results:**

Systematic review and expert panels, including our Lived Experience Advisory Panel, identified 66 candidate intervention components in five domains: Recruitment and Role Description of Peer Workers; Training for Peer Workers; Delivery of Peer Support; Supervision and Support for Peer Workers; Organisation and Team. A series of Local Advisory Groups were used to prioritise components and explore implementation issues using consensus methods, refining an intervention blueprint. A peer support handbook and peer worker training programme were produced by the study team and piloted in two study sites. Feedback workshops were held with peer workers and their supervisors to produce a final handbook and training programme.

The ENRICH trial is registered with the ISRCTN clinical trial register, number ISRCTN 10043328, and was overseen by an independent steering committee and a data monitoring committee.

**Supplementary Information:**

The online version contains supplementary material available at 10.1186/s13104-021-05735-0.

## Introduction

An increasing number of randomised controlled trials of one-to-one peer support in mental health services have taken place recently, with growing evidence of the effectiveness of peer support in improving self-reported recovery and empowerment outcomes [1]. Peer support in mental health services often involves peer workers—people with their own experiences of mental distress or using mental health services—employed and trained to provide various forms of support to people who have similar experiences of mental distress or mental health care. Other reviews have suggested that the benefits of peer support remain unclear where peer support is poorly described [2], limiting the usefulness of studies in informing policy and practice [3]. Qualitative research has indicated that the potential benefits of peer support can become diluted where key aspects are poorly defined, such as shared expectations of the peer worker role [4], clear peer worker role description [5], access to training and support [6], and preparation and training for clinical teams working alongside peer workers [7].

The importance of experiential knowledge of mental distress and of using mental health services in informing the development of peer support initiatives has been indicated [8]. Experiential knowledge can be defined as knowledge about the world acquired through everyday experiences of living in the world—including knowledge about our health and mental health [9]—in contrast to formal, technical knowledge learnt through education and professional training [10]. Health services research that is informed by experiential as well as clinical and academic forms of knowledge is often referred to as coproduced research [11, 12].

We conducted a randomised controlled trial of peer support for discharge from inpatient mental health care (ENRICH), to test the effectiveness of a peer worker intervention in reducing readmission post-discharge [13]. This paper reports the development of the ENRICH peer support intervention, with a focus on the role of experiential knowledge in a coproduced approach to research.

## Main text

### Methods

**Fig. 1:**
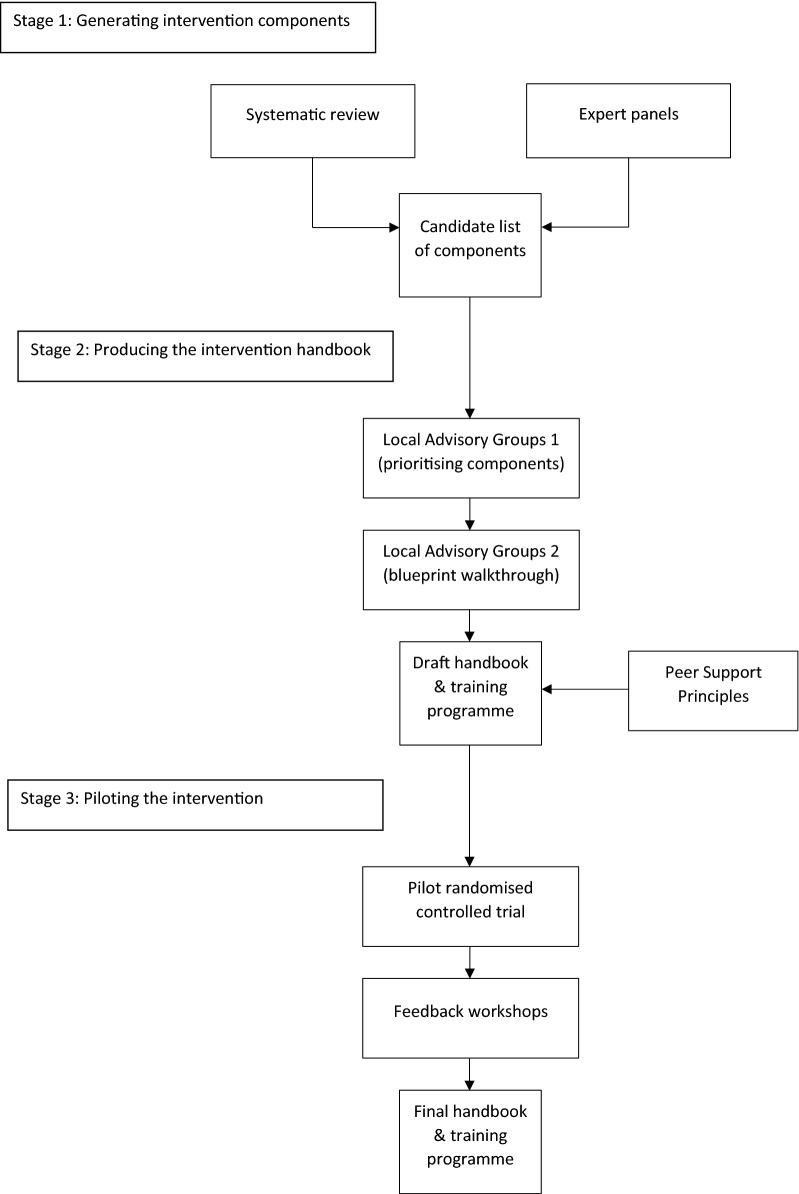
Stages of the intervention development process

The intervention was developed in three sequential stages, illustrated in Fig. 1—(1) generating intervention components; (2) producing the intervention handbook; (3) piloting the intervention—underpinned by a theoretical change model [14] and a principles framework for peer support [15] developed previously by the team.

#### Experiential knowledge in the development process

Several members of the research team identified as service user or survivor researchers, making explicit use of experiential knowledge in their work, or worked as peer workers. A Lived Experience Advisory Panel (LEAP) and Local Advisory Groups (LAG) at each study site also included people with experiences of using mental health services and peer support. The composition and role of these groups in the intervention development process is indicated in Table 1.

**Table 1:** Expertise in the intervention development process

	Research team	Lived Experience Advisory Panel	Local Advisory Groups
Number of people involved	14	13	48 (6 groups: average of 8 members per group)
Stage of process	1,2,3	1,2	2
Types of expertise (number of team members)	Service user/ survivor researchers (5) Clinical academics (2) Social scientists (2) Statistician (1) Peer workers (2) NHS managers (2)	Peer support leads in NHS and voluntary sector services Service user/ survivor researchers Peer workers/ peer supporters	Service users and carers Clinical team managers Mental health professionals Managers of voluntary sector services Peer workers

Stages of the intervention development process: 1 = generating intervention components; 2 = producing the intervention handbook; 3 = piloting the intervention

#### Stage 1: Generating intervention components

An intervention mapping approach was used to generate a list of components that might comprise the intervention [16]. We employed a systematic literature review and expert workshops to identify potential components. Components were given a short label and a descriptor, and mapped onto five domains: (1) Recruitment and Role Description; (2) Training; (3) Delivery; (4) Supervision and Support; (5) Organisation and Team. Where similar components were identified from different sources these were coded together.

*Systematic review* A systematic review of one-to-one peer support in mental health services was undertaken (International Prospective Register Of Systematic Reviews, identifier: CRD42015025621). The full method for the search is described in a systematic review and meta-analysis of randomised controlled trials of peer support [1]. For the purposes of intervention development, papers reporting studies of any design were included, from database inception until end of April 2015, where they reported description of intervention components. In addition, grey literature—unpublished evaluations and experiential testimonies—were identified using a snowball approach through emails to contacts known to be working in peer support. A member of the LEAP screened articles from the grey literature search with decisions checked by SG. Data detailing peer support components were extracted from included studies and coded to the five intervention domains.

*Expert workshops* Workshops were held with the LEAP and the research team to suggest potential components for the intervention. A third workshop was held with five members of the research team (JM, RF, MR, MU and SG) to consider how a taxonomy of Behaviour Change Techniques (BCT) [17] might be relevant to peer support in mental health services. Relevant elements of the taxonomy were mapped onto the five domains.

#### Stage 2: Producing the intervention handbook

*Prioritising components* LAGs were convened in each of six study sites (mental health Trusts; state service provider organisations). Names and descriptors of components identified in Stage 1 were printed on cards and prioritised using a closed card-sorting approach to consensus building [18]. In this case, LAGs prioritised each component by sorting them into a grid structured into the five domains, adding a maximum of five components to each domain. Components identified by three or more sources in Stage 1 (e.g. LEAP, team and review) were considered core to the intervention and already placed in their domains. Notes were made of LAG discussions, including the rationale for prioritising components. The research team produced a single grid based on the output from all LAGs. Components were discounted from further discussion if not prioritised by any LAGs, added as core components if prioritised by a majority of LAGs, or otherwise retained for further discussion.

*Producing and refining the intervention ‘blueprint’* Using output from the LAGs we produced a blueprint of the intervention in the form of a flow diagram, specifying the processes of recruiting peer workers, training, delivering the intervention, and support received by peer workers. The blueprint included all components retained for discussion so that local implementation issues could be considered.

In a second round of meetings LAGs were presented with the flow diagram and invited, using well-established talk-aloud approaches [19], to ‘walk through’ each stage of the flow diagram, discussing the sequencing or appropriateness of each component, reflecting on practicalities of implementing and supporting the intervention locally. Notes were made of each discussion.

*Drafting the intervention handbook* The output of LAG meetings was used to draft the ENRICH intervention handbook and peer worker training programme. Development of the intervention was also informed by our ‘peer support principles’ [15]. Further workshops with the LEAP and research team were held to inform writing the handbook and training content.

#### Stage 3: Piloting the intervention

A pilot randomised controlled trial of the intervention was conducted in two study sites to test feasibility of delivering trial procedures and implementation of the intervention [13]. Following the pilot, feedback workshops were held with the peer worker coordinators who trained and supervised peer worker teams at both sites, and the peer workers who had delivered the peer support at one site, exploring their experiences and views on what worked well and what might be improved about the training and other aspects of the peer support. Changes were made to the handbook and training programme, based on the feedback, following a further research team workshop.

### Results

#### Stage 1: Generating intervention components

A total of 3800 studies were identified in the literature search, of which 97 were included in the review, 85 peer-reviewed and 12 from grey literature (see Additional file 1). Components generated by the literature review and expert workshops were mapped onto the five intervention domains as shown in Additional file 2. Forty-four components were identified in the review (six from grey literature), 29 by the LEAP, 37 by the research team and six from the BCT workshop; a total of 66 distinct components once similar components were combined. The review contributed the most components to the recruitment and role description, training and delivery of peer support domains, with a number of qualitative studies offering detailed description of peer worker roles, training programmes and interventions. In contrast, peer support expertise in the research team and the LEAP contributed in particular to domains three and four (support for peer workers at individual and organisational levels), where this was less evident in the literature. Twelve core components, identified by three or more sources, are identified with an asterisk in Additional file 2.

#### Stage 2: Producing the intervention handbook

*Prioritising components* Following the first round of LAGs, six components were discounted, 19 added as core components (see Table 2), and 29 retained for further discussion.

**Table 2:** Core components of the intervention after first round of LAGs

1: Recruitment and role description	2: Training	3: Delivery of peer support	4: Supervision and support	5: Organisational and team support
Role description clearly describes peer’s approach around discovering and enabling service user’s strengths, empowering the individual to build their own support network post-discharge*	Training (and supervision) to include a focus on boundaries and managing relationships*	Peers to be part of formal discharge meeting/ care planning meetings where invited by the service user*	Regular group supervision for Peer Worker team from Peer Worker Coordinator	Ward and community teams—including managers—should receive a team preparation session co-delivered by peers working locally*
Role description to focus on identifying, signposting and, where requested by service user, accompanying to activities/ support/ opportunities using locally developed resource pack*	Training (and supervision) to include appropriate sharing of lived experience to role model post-discharge experience*	Peer to support/enable optional use of service user owned discharge plan, crisis plan and personal recovery plan*	Appropriate support always accessible when supervision (Peer Worker Coordinator) is unavailable	Peer workers require a ‘team base’
Person specification to include the ability to reflect on personal experiences	Training to be co-delivered by experienced peer workers*	Preparation for ending the support to be on the agenda from the outset*		Peer Worker Coordinator, and where possible Peer Workers, should visit wards/teams at part of set up
Peer leadership in recruitment and interview process essential	Training to cover key communication and supporting self-management skills*	Initial contact on the ward to focus on listening to the service user and relationship building		Peer support for discharge should be embedded in the Trust’s strategies
Role description to clearly indicate expectations of the role, with service user to be provided with information sheet clearly indicating expectation of the peer support role	Training structured around core set of values-based competencies*	First meeting between peer worker and service user post-discharge should be in addition to follow-up by community team		Clinical team preparation sessions should involve team members identifying the assets that peer workers will bring
	Training (and supervision) to include comprehensive coverage of working with risk and safety*			Employment of Peer Workers on the workforce should be integrated into HR policies
	Training to include standard Trust induction			
	Training to include locally led ‘community asset mapping’ session			
	Existing locally developed training sessions included in peer worker training where these cover required skills/competencies			
	Training (and supervision) to include a focus on keeping yourself well and safe at work			
	Training to include specific focus on experience of the discharge ‘transition’			
	Training to include cultural competence, gender, religious, cultural issues etc			
	Training (and supervision) for Peer Worker in discussing difficult issues			

*Original core component identified in stage 1

*Producing and refining the intervention blueprint* The flow diagram used in the walkthrough exercises in the second round of LAGs is shown in Additional file 3.

*Drafting the intervention handbook* A detailed handbook was produced specifying a full set of procedures defining peer worker and the peer worker coordinator roles, recruitment process, training, support and supervision for peer workers, and how the peer support is delivered in hospital and in the community (see Table 3).

**Table 3:** Content of ENRICH peer support handbook

Chapter	Content
1.0 What is ENRICH?	1.1 Why peer support for discharge? 1.2 What is the ENRICH project and why do we need it? 1.3 The ENRICH research team
2.0 Peer support for discharge—a principles-based approach	2.1 Developing the principles framework 2.2 Applying the framework in ENRICH peer support for discharge
3.0 Developing the ENRICH peer support handbook	3.1 Generating ideas 3.2 Arriving at a consensus 3.3 Piloting the handbook
4.0 The ENRICH peer worker role	4.1 Role description 4.2 Person specification 4.3 Working pattern and flexibility 4.4 Remuneration
5.0 The Peer Worker Coordinator role	5.1 Role description and person specification 5.2 Duties and responsibilities 5.3 Remuneration 5.4 Support and supervision for the Peer Worker Coordinator 5.5 Cover in the absence of the Peer Worker Coordinator
6.0 Peer worker recruitment process	6.1 Pathway 1—advertising and recruiting new peer workers 6.2 Pathway 2—assigning peer workers from existing peer workforce 6.3 Advertising the role 6.4 Information event and pre-training meeting 6.5 Role of training assessment in recruitment process 6.6 Job application and interview 6.7 Employment and welfare support 6.8 Appointment to role/appointment to reserve 6.9 DBS checks and Occupational Health 6.10 Recruitment numbers
7.0 The ENRICH training programme	7.1 Structure of training programme (a principles-based approach) 7.2 Delivery of training (role of the Peer Worker Coordinator) 7.3 Content of training sessions 7.4 Use of local training modules 7.5 Feedback and reflection 7.6 Assessment methods 7.7 Site visits
8.0 Accessing patient notes	8.1 Peer workers with access to electronic patient notes 8.2 Peer workers without access to electronic patient notes
9.0 Induction	9.1 Peer worker team induction 9.2 NHS induction 9.3 Ward visits and shadowing
10.0 Preparing NHS teams	10.1 Ward and community team preparation workshops
11.0 Supervision and support for peer workers	11.1 Group supervision 11.2 Individual supervision 11.3 Absence of Peer Worker Coordinator 11.4 Risk, safety and handover 11.5 Access to peer support for peers 11.6 Peer worker wellbeing plan 11.7 Team base
12.0 Pairing of peer workers and service users	12.1 The research process (allocation to peer support) 12.2 Peer Worker Coordinator preference meeting with service user
13.0 Delivery on the ward	13.1 First meeting 13.2 Frequency, location and duration of meetings 13.3 Use of service user-owned discharge plan 13.4 Peer worker involvement in formal discharge planning 13.5 Peer worker relationship to ward team 13.6 Risk, safety and handover
14.0 Delivery in the community	14.1 First meeting post-discharge 14.2 Frequency, location and duration of meetings 14.3 Lone/home working 14.4 Telephone and social media contact 14.5 Use of service user-owned plans and tools 14.6 Accompanying 14.7 Peer worker relationship to community mental health teams 14.8 Ten week step down 14.9 Endings 14.10 ENRICH Peer Worker Code of Ethics 14.11 Readmission to hospital during community-based peer support
15.0 Peer worker absence	15.1 Short term cover (within team) 15.2 Long term cover (reserve peer workers) 15.3 Support and induction for reserve peer workers

An eight-day, manualised training programme was developed, underpinned by ten knowledge and skills sets derived from components prioritised to the training domain in Stage 2, and the peer support principles [15]. The resulting training matrix (Additional file 4) guided writing and development of training materials. Each training day comprised session plans, slides, handouts, exercises and other materials.

#### Stage 3: Piloting the intervention

Five peer worker coordinators involved in delivering training at both pilot sites attended a feedback workshop, with one peer worker coordinator providing individual feedback. Four of five peer workers at one site attended a workshop. Feedback was used to make amendments to the training and aspects of the handbook on recruitment of peer workers and post-training support (see Additional file 5).

### Discussion

This paper reports a rigorous process of intervention development, resulting in production of a detailed handbook and manualised training programme for peer support for discharge from inpatient mental health care. We used an intervention mapping approach [16] that incorporates existing evidence and expert opinion, and in particular, experiential knowledge of using mental health services and peer support [8]. The Lived Experience Advisory Panel, service user researchers and peer workers on the research team helped generate the content of the intervention (Table 2), shaping the intervention in a way that would have been missing if experiential knowledge had not been foregrounded in the development process. Local Advisory Groups at each study site (comprising service users and peer workers as well as clinicians and health service managers) were involved in prioritising intervention components and identifying variation to the intervention appropriate to local service environments. It has been suggested that involving a full range of stakeholders in the development of psychosocial interventions in mental health improves engagement with, and fidelity of, interventions [20], and that the active involvement of people who have used mental health services in undertaking mental health research readies organisations to implement experiential knowledge into practice [21]. Further research is needed to ascertain whether coproducing our intervention in this way improved fidelity [22] and engagement in the trial.

## Limitations

Development closely followed the Medical Research Council complex interventions guidance [23], including: being grounded in a coherent theoretical framework and change model; informed by the existing evidence base; fully described to aid implementation and replication; designed with consideration of real-world implementation issues in mind. Reporting of the development process paid attention to most domains of the GUIDED approach [24], and in particular to ‘stakeholder contribution’ through our focus on incorporating experiential knowledge into the process. We did less well in considering heterogeneity in the population targeted by the intervention—psychiatric inpatients—and therefore possible differential effects on subgroups. Our intervention is deliberately transdiagnostic [13], and there is a lack of research exploring differing processes or impact of peer support in specific diagnostic groups [25]. In addition, while it is well known that there are inequalities in access, experiences and outcomes of mental health care between different ethnic groups [26], understanding of peer support in different cultural contexts is limited [27]. Our trial is designed to explore subgroup effects including diagnostic group and ethnicity [13], but further research will be necessary to explore if and how the intervention might benefit from adaptations to different groups of people who might be offered peer support.

## Supplementary Information

**Additional file 1.** Flow diagram of inclusion of studies in the systematic review.

**Additional file 2.** Candidate components for the peer support intervention.

**Additional file 3.** Flow diagram used for walkthrough exercise in second round of Local Advisory Groups.

**Additional file 4.** The ENRICH training matrix.

**Additional file 5.** Feedback from intervention pilot workshops.

## Data Availability

The datasets used and analysed during the current study, and the handbook and training materials are available from the corresponding author on reasonable request.

## Abbreviations

BCT: Behavioural Change Techniques
DBS: Disclosure & Barring Service
LAG: Local Advisory Group
LEAP: Lived Experience Advisory Panel
NIHR: National Institute for Health Research
NHS: National Health Service
