# Supplementary figures and images for: Development of a peer support intervention to improve the experience and outcomes of discharge from inpatient mental health care: the role of experiential knowledge in a coproduced approach
Source: BMC Res Notes. 2021 Aug 21;14:320. doi: 10.1186/s13104-021-05735-0 (PMC8379721; doi:10.1186/s13104-021-05735-0)

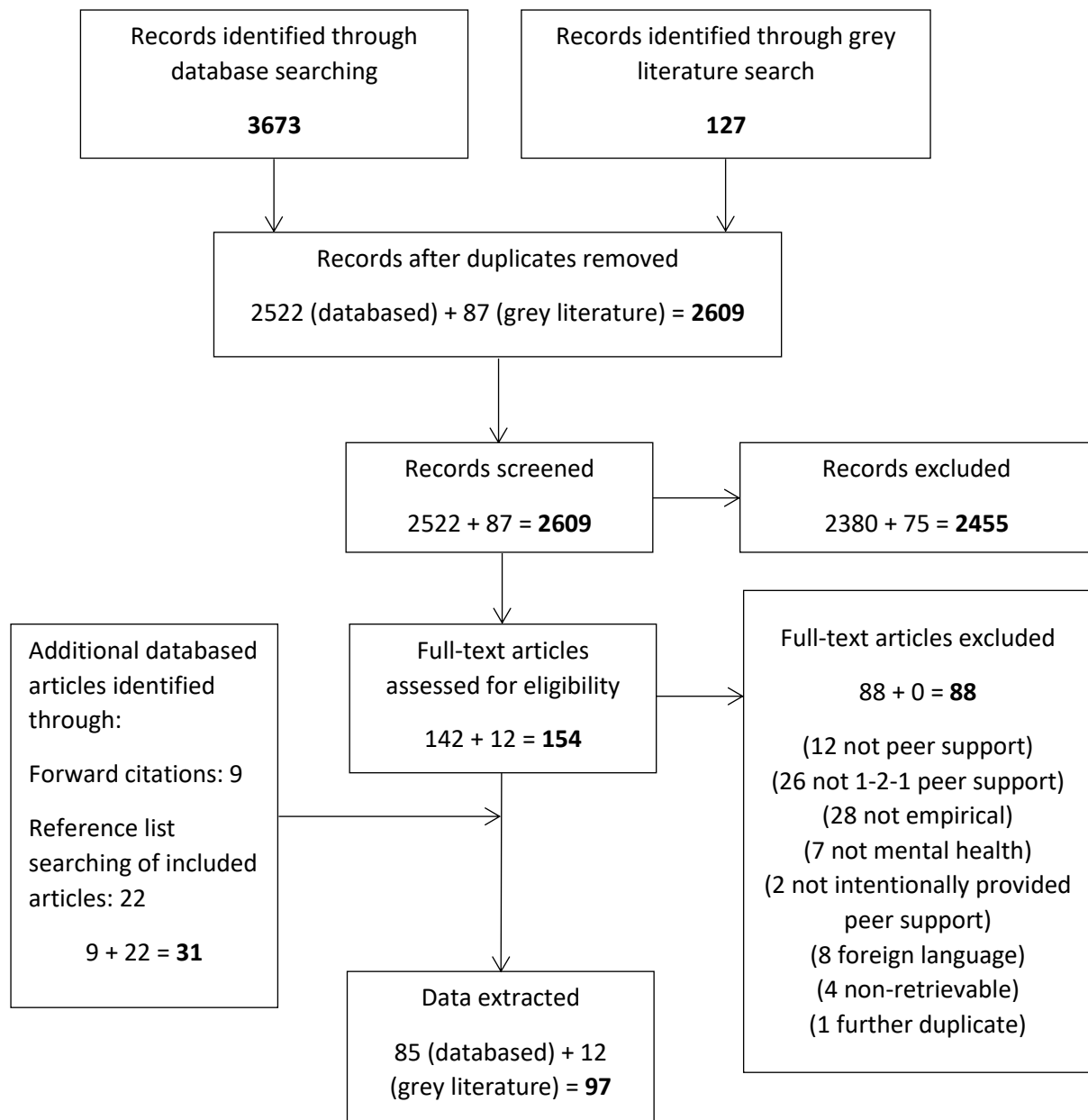

Supplement: Supplementary file 1 — Additional file 1. Flow diagram of inclusion of studies in the systematic review. [file 13104_2021_5735_MOESM1_ESM.pdf]
