# Supplementary material for: Development of a peer support intervention to improve the experience and outcomes of discharge from inpatient mental health care: the role of experiential knowledge in a coproduced approach
Source: BMC Res Notes. 2021 Aug 21;14:320. doi: 10.1186/s13104-021-05735-0 (PMC8379721; doi:10.1186/s13104-021-05735-0)
Supplement: Supplementary file 2 — Additional file 2. Candidate components for the peer support intervention. [file 13104_2021_5735_MOESM2_ESM.pdf]

Domain	Label	Descriptor	Source
Domain 1: Recruitment and role description	Person specification to include definition of lived experience		LEAP
	Person specification to include having been an inpatient	Desirable rather than essential criteria	LEAP
	Person specification to include the ability to reflect on personal experiences	Personal experiences of mental distress, recovery, and giving and receiving support	LEAP, Review
	Peer leadership in recruitment and interview process essential	Ideally should be the Peer Worker Coordinator	LEAP, Review
	Person specification to include ability to record and report		LEAP, Review
	Interview to ask how working as a peer worker fits with future career aspirations		LEAP
	Person specification to include knowledge of living and using services locally		LEAP
	Recruitment process to take the form of a whole day event or open day	Potential peer workers to engage in observed, scenario-based group and pairs work etc during the day, not just a one-to-one interview	Team, Review
	Recruitment is to training (not to job)	Sufficient people recruited to training to allow for drop-out/ people not moving into a job, plus a reserve (approx. double number required for posts)	Team, Review
	Person specification to include having completed recovery training/ have a recovery plan	Not essential, but to be a requirement of training if not already in place	Team
	*Role description clearly describes peer's approach around discovering and enabling service user's strengths, empowering the individual to build their own support network post-discharge	Note the peer should not become a substitute for that network for a limited period (linked to competencies in training)	LEAP, BCTs, Review
	*Role description to focus on identifying, signposting and, where requested by service user, accompanying to activities/ support/ opportunities using locally developed resource pack	Resource pack linked to local resource mapping session in training; to include a) things the individual did before admission, b) new things; peer to have sufficient flexibility in the role to attend activities as appropriate	LEAP, Team, Review
	Role description to clearly indicate expectations of the role, with service user to be provided with information sheet clearly indicating expectation of the peer support role	Noting that role is not about the peer being an advocate or a generic support worker	LEAP
	Person specification to include strong interpersonal skills, interest in helping others etc. to be tested at interview		Review
	Recruitment by advertisements in services/ organisations that might employ peers		Review
	Letters of reference required at recruitment	Can be character references in the absence of work references	Review

	Homework and/ or tests (quizzes) as part of the assessment process		Review (grey lit.)
	Pre-screening telephone interview prior to invitation to recruitment day	Assessing how comfortable people are talking about their mental health, scenarios around providing support etc	Review
Domain 2: Training	Training to include work-based placements	Including working alongside existing peers	Team, Review
	Training to include standard Trust induction	Note: this might include Trust's standard breakaway training etc. although peers would not be required to use this as part of their role	Team
	Training to include locally led 'community asset mapping' session		Team, LEAP
	Training to incorporate locally developed/ delivered sessions where these cover required skills/ competencies		LEAP, Review
	Training to have at least some minimal level of accreditation		LEAP
	*Training (and supervision) to include a focus on boundaries and managing relationships	For example, on blurred friendship line, especially where there is a pre-existing friendship or there is an attraction between peer and service user	LEAP, Team, Review
	Training to include session on preparing for the workplace	For example, working on the ward, plus basics around use of phones, office etc.	Team, Review
	Training (and supervision) to include a focus on keeping yourself well and safe at work	Including the welling and mental health of the peer	Team, Review
	*Training (and supervision) to include appropriate sharing of lived experience to role model post-discharge experience	Linked to competencies in training	Team, BCTs, Review, LEAP
	*Training to be co-delivered by experienced peer workers	In similar roles (if not the same)	LEAP, Team, Review
	Training to include cultural competence, gender issues etc		Review
	Training to include competence around addressing stigma		Review
	Training (and supervision) for peer in discussing difficult issues	E.g. suicidality, self-harm, drug taking, sexual abuse etc	Team
	*Training to cover key communication and supporting self-management skills	Peer has a range of core skills - e.g. reflective listening, summarising, goal-planning etc – to use in addition to sharing of lived experience	LEAP, BCTs, Review, Team
	*Training structured around core set of values-based competencies	Exercises and assessments to cover each competency (e.g. mutuality, reciprocity, non-directive working, validating etc)	Team, BCTs, Review
	Assessments during and at end of training around each key competency	Including role play type assessments plus readiness to work interview; used to decide who moves from training to full or reserve role	Team, Review

	*Training (and supervision) to include comprehensive coverage of working with risk and safety	Including decision making about what and when to handover to clinical team; if Peer Worker Coordinator does not have clinical experience appropriate clinical link person should be identified in the Trust	LEAP, Team, Review
	Training (and supervision) to include a focus on keeping yourself well and safe at work	Including the welling and mental health of the peer	Team, Review
Domain 3: Delivery of peer support	Peers to accompany people on home visits/ leave while still in hospital, and meet people in their homes while living in the community	Where possible, to support reconnecting with people and places; only where a preference for that is expressed by the service user; must conform to employer's lone working policy	Team, LEAP
	*Peers to be part of formal discharge meeting/ care planning meetings where invited by the service user	To accompany, support and enable (not to advocate)	LEAP, Team, Review
	Initial contact on the ward to focus on listening to the service user and relationship building	Focus on building trust, building a rapport, not on the peer telling their story (appropriate sharing of lived experience to make a connection)	LEAP, Team
	*Peer to support/ enable optional use of service user owned discharge plan, crisis plan and personal recovery plan	Discharge tool to focus on what service user wants/ does not want post-discharge, rather than prescriptive planning of activities; all tools, can use locally available or any suitable strengths-based tools	LEAP, Team, BCTs, Review
	First meeting with peer post-discharge should be in addition to follow-up by community team	Not instead of, or part of that statutory meeting	LEAP
	Clear guidance for peers on telephone, text, email and social media contact		Team
	Matching of peers to service user by key demographics, experience of services and/ or hobbies & interests	As far as possible given size of peer worker team	Review
	Peers direct service users to appropriate online resources	Including information sites, mental health discussion forums, online self-management/ psycho-social education etc	Review
	Peer encourages involvement of 'significant others' in any personal/ recovery/ crisis planning		Review
	Peers will only write to (Trust) clinical notes what is agreed with service user		Team, Review
	Health lifestyle and health promotion included in training/ delivery		Review (grey lit.)
	Medication and symptom management included in training/ delivery		Review (grey lit.)
	Messages linked to recovery planning/goal setting sent by peer worker using text or email		Review (grey lit.)

	Peer Worker to help service user to complete self-assessment and monitoring tools		Review (grey lit.)
	*Preparation for ending the support to be on the agenda from the outset	Contact reducing from weekly to fortnightly towards the end of peer support, training and supervision to include working with attachment, therapeutic relationship, endings etc (including ending of individual sessions as well as support relationship)	LEAP, BCTs, Team, Review
	The Peer Worker fulfils a 'navigator' role, helping service user assess health and social care needs, identify providers, make appointments and accompany as necessary		Review (grey lit.)
Domain 4: Support and supervision (Note: a number of components included in training domain also apply to supervision)	Regular peer to peer support meeting	Sharing of experiences, strategies etc by peers, plus opportunity to recognise successes in the workplace etc	LEAP, Team
	Group supervision for peers from Peer Worker Coordinator	With option of one-to-one supervision always available	Team
	Appropriate support always accessible when supervision (Peer Worker Coordinator) is not available	By phone or face-to-face, including when Peer Worker Coordinator is unavailable, so peer is never left isolated holding a difficult issue	LEAP, Review
	Supervision from someone with lived experience essential	Included in the person specification for the Peer Worker Coordinator (note: if a suitable individual is not available locally for the role external supervision from a peer should be resourced, in addition to line management from within the Trust)	LEAP, Team
	Peer Worker Coordinators to undertake their own recovery planning		Team
	Peer Worker Coordinators should have access to learning sets with people in the same/ equivalent roles	Either locally or more widely if necessary	Team
	Development programme in place for people moving into reserve roles	To include, at a minimum, a monthly development meeting plus opportunities for placements in, for example, local recovery college and help in applying for other (voluntary or paid) peer roles	Team
Domain 5: Team and organisational support	Peer Workers require a 'team base'	Including access to office space; somewhere they feel safe and can take time out, meet and build supportive relationships with colleagues (flexibility locally re most appropriate location)	Team, Review
	Links to good employment support services	Can be in the Trust or local voluntary sector provider	Team, LEAP
	*Ward and community teams – including managers – should receive a	Focus on role of peer support alongside current service delivery	Team, LEAP, Review

team preparation session co-delivered by peers working locally		
Peer Worker Coordinator, and where possible peers, should visit wards/ teams as part of set up	To establish communication, explain processes, address expectations etc.	Team, Review
Peer support for discharge should be embedded in Trust's recovery strategy, strategic development, planning documents etc		Team, Review
Clinical team preparation sessions should involve team members identifying the assets that peer workers will bring	Not a teaching session; use of appropriate tools	Team
Employment of peers on the workforce should be integrated into HR policies		Review, Team

*Core components identified in three or more sources
