# Supplementary material for: Development of a peer support intervention to improve the experience and outcomes of discharge from inpatient mental health care: the role of experiential knowledge in a coproduced approach
Source: BMC Res Notes. 2021 Aug 21;14:320. doi: 10.1186/s13104-021-05735-0 (PMC8379721; doi:10.1186/s13104-021-05735-0)
Supplement: Supplementary file 3 — Additional file 3. Flow diagram used for walkthrough exercise in second round of Local Advisory Groups. [file 13104_2021_5735_MOESM3_ESM.pdf]

Role description (inc.)
1) Defines expectations & boundaries of the role
2) Describes a strengths-based, network building approach
3) Describes an enabling (inc. accompanying) & signposting, rather than support worker function

Person specification
1) Ability to reflect on personal experiences (essential)
2) Lived experience of inpatient care (desirable)
3) Lived experience of mental distress AND of having used mental health services (essential)
4) knowledge of local community (desirable)

Sites to adopt either entry route 1 or 2 to training/ role

1. Advert placed in local services & voluntary sector/ community partners – advertised as training opportunity with possibility to move info paid peer worker role

2. Suitable individuals identified among existing peer team for streaming to ENRICH role

Written expression of interest

Half day information event – one-to-one discussion assessing suitability/ motivation against criteria – DBS* check initiated for those proceeding

Training to be structured around core values-based competencies, including a focus on:

1) Discharge transition
2) Keeping yourself well at work (inc asking for support)
3) Boundaries & managing relationships (inc endings, ‘over-involvement’)
4) Working with risk & safety
5) Local ‘community asset mapping’ + local mental health services (how they work)
6) Discussing difficult issues (suicidality, medication)
7) Cultural competency
8) Communication & self-management skills
9) Sharing lived experience

Training to be led by Peer Worker Coordinator and co-delivered by peers
Training to cover preparing an NHS Jobs application, employment planning etc
Development of wellbeing plan (including treatment preferences)

People with previous peer training (inc route 2) required to undertake all sessions and assessments as a team and to explore discharge specific application

Locally developed sessions substituted where core skills & competencies are sufficiently covered

Opportunities for ward and community visits where no/ little previous experience

Operational Policy (NHS employer)/ Collaboration Agreement (vol sector employer) setting out: risk and handover; complaints & disciplinary; lone/ home working; note writing/ information access; NHS training/ induction requirement etc.

*DBS check = Disclosure & Barring Service (criminal record) check

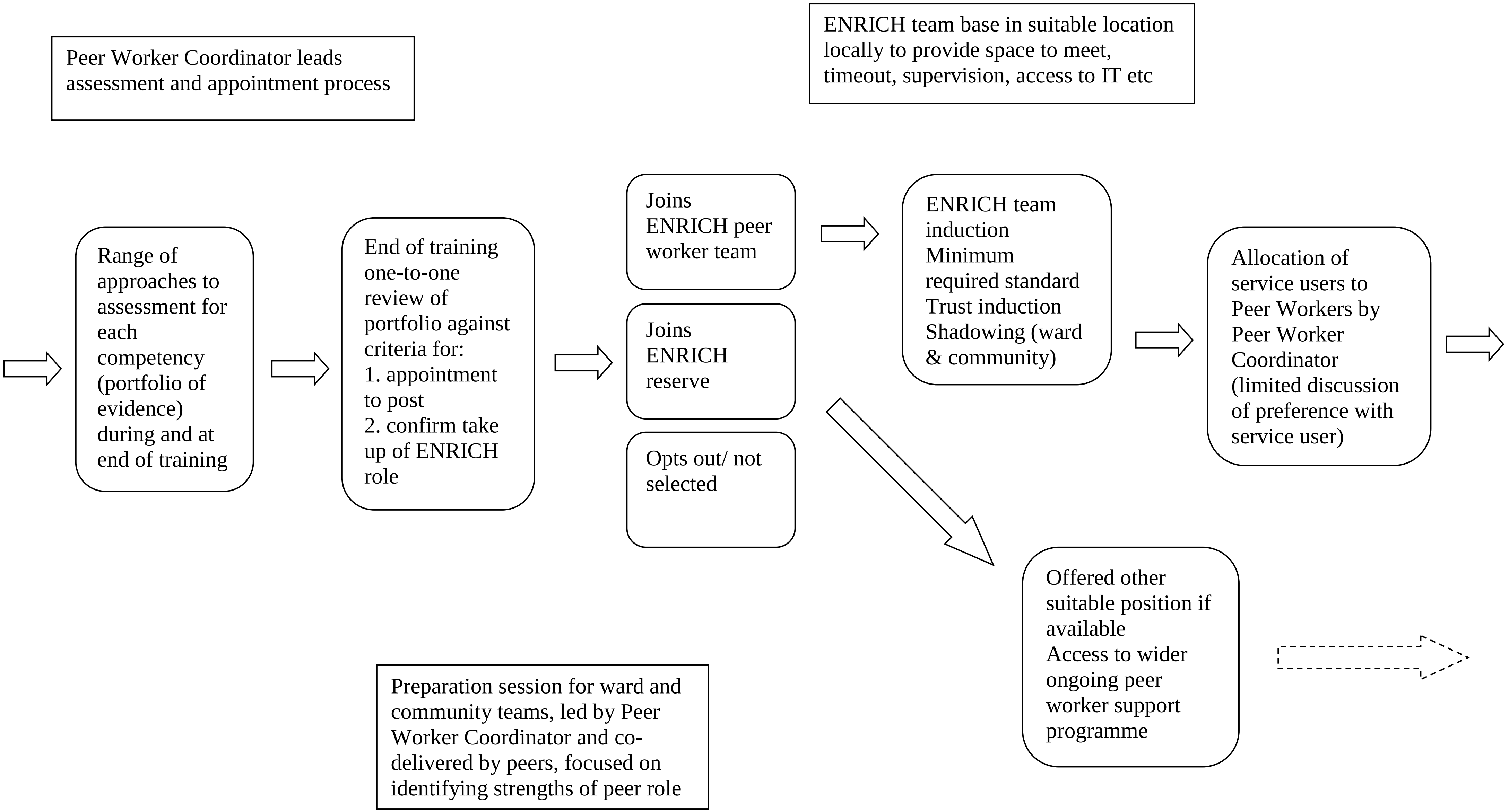
Peer Worker Coordinator leads assessment and appointment process
ENRICH team base in suitable location locally to provide space to meet, timeout, supervision, access to IT etc
Range of approaches to assessment for each competency (portfolio of evidence) during and at end of training
End of training one-to-one review of portfolio against criteria for:
1. appointment to post
2. confirm take up of ENRICH role
Joins ENRICH peer worker team
Joins ENRICH reserve
Opts out/ not selected
ENRICH team induction
Minimum required standard
Trust induction
Shadowing (ward & community)
Allocation of service users to Peer Workers by Peer Worker Coordinator (limited discussion of preference with service user)
Offered other suitable position if available
Access to wider ongoing peer worker support programme
Preparation session for ward and community teams, led by Peer Worker Coordinator and co-delivered by peers, focused on identifying strengths of peer role

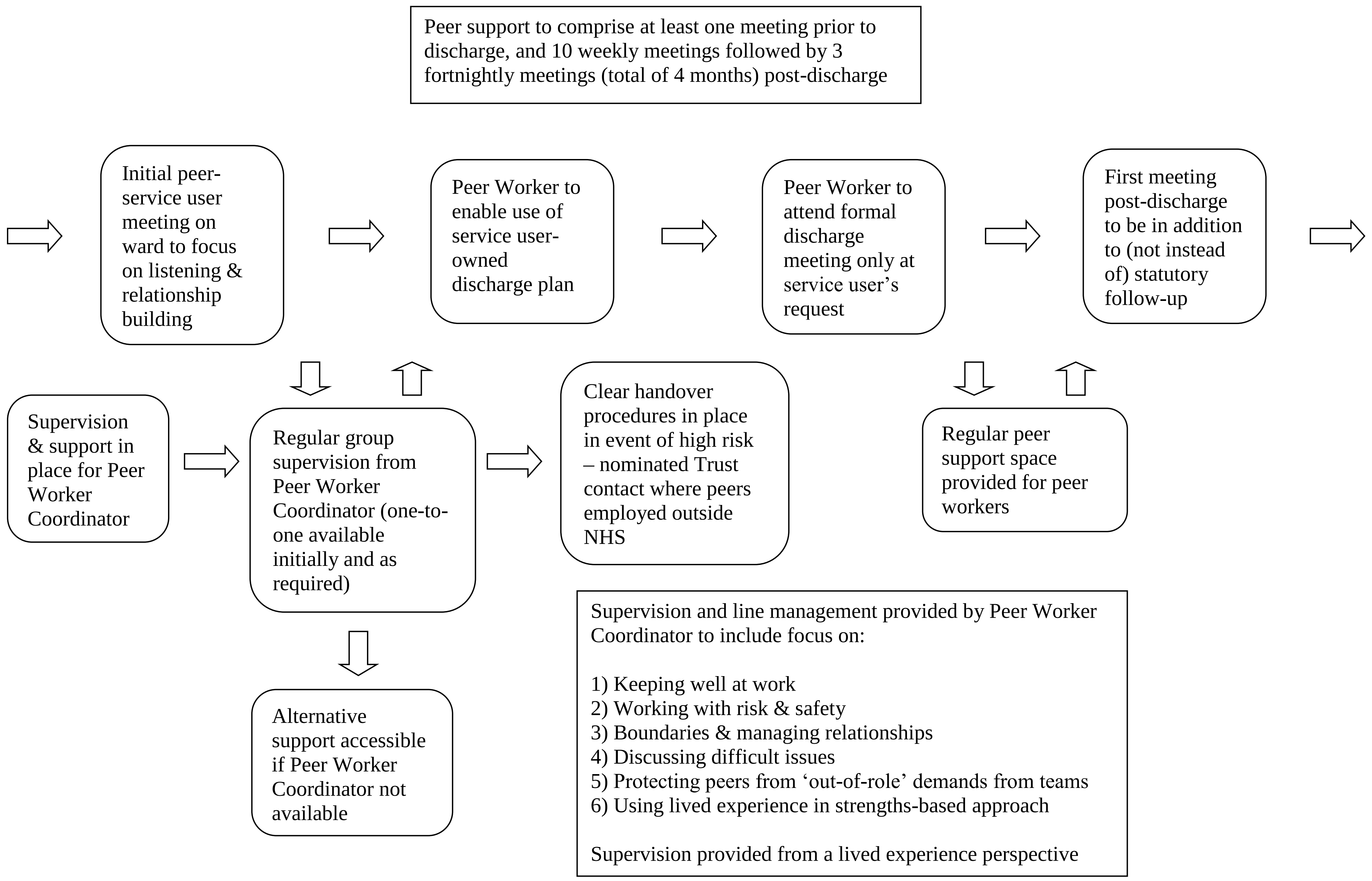
Peer support to comprise at least one meeting prior to discharge, and 10 weekly meetings followed by 3 fortnightly meetings (total of 4 months) post-discharge
Initial peer-service user meeting on ward to focus on listening & relationship building
Peer Worker to enable use of service user-owned discharge plan
Peer Worker to attend formal discharge meeting only at service user's request
First meeting post-discharge to be in addition to (not instead of) statutory follow-up
Supervision & support in place for Peer Worker Coordinator
Regular group supervision from Peer Worker Coordinator (one-to-one available initially and as required)
Clear handover procedures in place in event of high risk – nominated Trust contact where peers employed outside NHS
Regular peer support space provided for peer workers
Alternative support accessible if Peer Worker Coordinator not available
Supervision and line management provided by Peer Worker Coordinator to include focus on:
1) Keeping well at work
2) Working with risk & safety
3) Boundaries & managing relationships
4) Discussing difficult issues
5) Protecting peers from 'out-of-role' demands from teams
6) Using lived experience in strengths-based approach
Supervision provided from a lived experience perspective

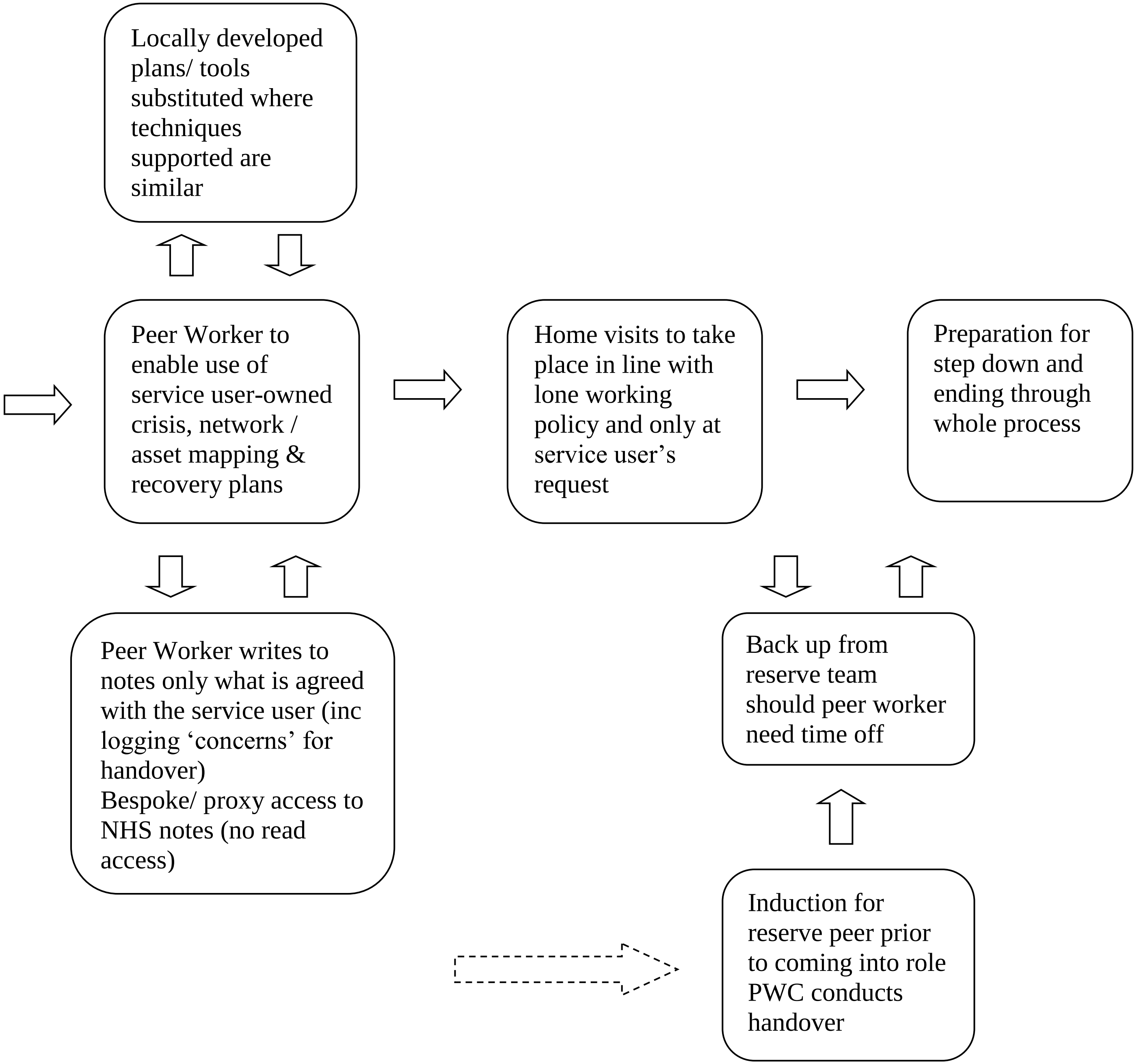
Locally developed plans/ tools substituted where techniques supported are similar
Peer Worker to enable use of service user-owned crisis, network / asset mapping & recovery plans
Home visits to take place in line with lone working policy and only at service user's request
Preparation for step down and ending through whole process
Peer Worker writes to notes only what is agreed with the service user (inc logging 'concerns' for handover)
Bespoke/ proxy access to NHS notes (no read access)
Back up from reserve team should peer worker need time off
Induction for reserve peer prior to coming into role PWC conducts handover
