# Supplementary material for: Development of a peer support intervention to improve the experience and outcomes of discharge from inpatient mental health care: the role of experiential knowledge in a coproduced approach
Source: BMC Res Notes. 2021 Aug 21;14:320. doi: 10.1186/s13104-021-05735-0 (PMC8379721; doi:10.1186/s13104-021-05735-0)
Supplement: Supplementary file 4 — Additional file 4. The ENRICH training matrix. [file 13104_2021_5735_MOESM4_ESM.pdf]

<table>
<tr><td>Understanding the discharge transition & mental health services</td><td colspan="5">Session 1 – Introduction to peer support & the ENRICH peer worker role</td></tr>
<tr><td>Sharing lived experience</td><td colspan="5">Session 2 – Sharing lived experience</td></tr>
<tr><td>Using communication skills</td><td colspan="5" rowspan="2">Session 3 – Communication skills & strengths-based approaches</td></tr>
<tr><td>Using strengths-based approaches</td></tr>
<tr><td>Working with boundaries & relationships</td><td colspan="5">Session 4 – Working with boundaries & relationships</td></tr>
<tr><td>Cultural awareness & understanding diversity</td><td colspan="5">Session 5 – Cultural awareness & diversity in peer support</td></tr>
<tr><td>Local & individual ‘asset mapping’</td><td colspan="5">Session 6 – Individual & community asset mapping</td></tr>
<tr><td>Discussing difficult issues</td><td colspan="5">Session 7 – Discussing difficult issues</td></tr>
<tr><td>Keeping well at work</td><td colspan="5" rowspan="2">Session 8 – Working with risk & staying well at work</td></tr>
<tr><td>Working with risk and safety</td></tr>
<tr><td rowspan="2">Knowledge & skills sets
Principles</td><td>1. Safe trusting relationships built on shared lived experience</td><td>2. Mutuality & reciprocity</td><td>3. Applying experiential knowledge</td><td>4. Leadership, choice & control</td><td>5.Discovering strengths & building connections</td></tr>
<tr><td colspan="5">Peer support should respect and support the full diversity of experiences, language, culture, identity and background that people bring, found in the range of communities with which they identify and belong</td></tr>
</table>
