# Supplementary material for: Development of a peer support intervention to improve the experience and outcomes of discharge from inpatient mental health care: the role of experiential knowledge in a coproduced approach
Source: BMC Res Notes. 2021 Aug 21;14:320. doi: 10.1186/s13104-021-05735-0 (PMC8379721; doi:10.1186/s13104-021-05735-0)
Supplement: Supplementary file 5 — Additional file 5. Feedback from intervention pilot workshops. [file 13104_2021_5735_MOESM5_ESM.pdf]

	Peer Worker Coordinator feedback	Resulting changes to training/ handbook	Peer Worker feedback
Pre-training	Need for preliminary meeting before training begins	Introductory content of first training session improved	Open Day was very useful, importance of advertising through all relevant local services/ organisations<br>Informal pre-training one-to-one discussion with peer worker coordinator felt like an interview; however helpful that it was presented as informal and good interview practice
Training	Training was rewarding but exhausting, with not enough time for consolidation<br>Sometimes too much content, language could be too academic<br>Experiential approach to training worked well<br>Peer worker coordinators also felt like they learnt from training<br>Importance of at least one trainer have experience of peer work<br>Need to improve training materials for diversity session (cultural competency self-assessment tool) with more space for self-reflection and thinking about stigma<br>Trainees became adept at supporting each other/ developing their own peer support network<br>Trainees reported that they could feel like they were being judged during role plays (sometimes they were performing rather than practicing)<br>Assessment matrix a useful tool<br>Purpose and usefulness of assessment and trainees' reflective logs unclear<br>Lack of feedback space, perhaps introduce an additional session part way through training<br>Training material for session on boundaries was over complicated not allowing as much space for trainees to learn from each other<br>Clear guidance needed on importance of working with others/ handover in session about suicidality	Option to lengthen training to 8 sessions once a week over 8 weeks (from twice a week over 4 weeks)<br>Content and language reviewed and reduced/ amended as appropriate<br>Alternative cultural competency tool sourced<br>A lighter touch approach taken to assessment; assessment of skill sets as a whole, rather than individual exercises<br>New, supplementary trainer and trainee handbooks produced, including clarification of purpose of assessment, role plays and reflective logs<br>An additional one-to-one feedback session introduced for peer workers in new 'rest week' after session 4<br>Training material for boundaries session simplified with less information content and more space of experiential exercises<br>Amendments made to content as necessary	Content and structure of training good but intense<br>Role plays and other exercises good, sharing experiences and learning from others very useful<br>Trainers with a mix of expertise good<br>Reflective logs very useful but greater clarity needed around purpose
Post-training	Need for more information for trainees on 'what happens next' after training<br>Once peer workers were working, need for ongoing training around working alongside clinical teams	Improved guidance in handbook on supporting trainees at the end of training<br>Guidance included in handbook<br>Guidance added to handbook covering on-the-job training, to be identified with peer workers once in post	Need for more communication between training and starting to work; employment support invaluable<br>Induction very useful but a full range of practical issues should be covered; team base to be properly equipped<br>Training needed on drug and alcohol support, housing and debt
